# Contrasting interferon-mediated antiviral responses in human lung adenocarcinoma cells

**DOI:** 10.1128/jvi.00469-25

**Published:** 2025-05-28

**Authors:** Matthew Esparza, Sara S. El Zahed, Umut Karakus, Hanspeter Niederstrasser, Boning Gao, Kimberly Batten, Jerry W. Shay, Bruce Posner, Fred R. Hirsch, Luc Girard, Lily Jun-shen Huang, John Minna, Adolfo García-Sastre, Beatriz M. A. Fontoura

**Affiliations:** 1 Department of Cell Biology, University of Texas Southwestern Medical Center 12334, Dallas, Texas, USA; 2 Department of Microbiology, Icahn School of Medicine at Mount Sinai 5925, New York, New York, USA; 3 Global Health and Emerging Pathogens Institute, Icahn School of Medicine at Mount Sinai 5925, New York, New York, USA; 4 Department of Biochemistry, University of Texas Southwestern Medical Center 12334, Dallas, Texas, USA; 5 Department of Pharmacology, University of Texas Southwestern Medical Center 12334, Dallas, Texas, USA; 6 Hamon Center for Therapeutic Oncology Research, University of Texas Southwestern Medical Center 12334, Dallas, Texas, USA; 7 Center for Thoracic Oncology, Icahn School of Medicine at Mount Sinai 5925, New York, New York, USA; 8 The Tisch Cancer Institute, Icahn School of Medicine at Mount Sinai 5925, New York, New York, USA; 9 Department of Pathology, Molecular and Cell-Based Medicine, Icahn School of Medicine at Mount Sinai 5925, New York, New York, USA; 10 Departments of Internal Medicine and Pharmacology, University of Texas Southwestern Medical Center 12334, Dallas, Texas, USA; 11 Department of Medicine, Division of Infectious Diseases, Icahn School of Medicine at Mount Sinai 5925, New York, New York, USA; 12 The Icahn Genomics Institute, Icahn School of Medicine at Mount Sinai 5925, New York, New York, USA; University of North Carolina at Chapel Hill, Chapel Hill, North Carolina, USA

**Keywords:** influenza, lung cancer, interferons, viral entry

## Abstract

Lung cancers develop from lung epithelial cells after a series of genetic and epigenetic changes, and these cells are major sites of influenza virus infection. Thus, we explored how changes found in patient-derived lung cancer cell lines impacted influenza virus replication and identified two lines with opposite responses to influenza A viral infection. We show that the NCI-H820 lung adenocarcinoma (LUAD) is resistant to influenza A virus and VSV infection, while LUAD line NCI-H322 is highly susceptible to infection by both viruses. H322 cells have a homozygous deletion in a region of chromosome 9 encoding IFNαgenes, IFNβ1, IFNω1, and IFNε genes, leading to downregulation of immune response and high infection rates. In contrast, the resistant H820 cell line has three copies of these same interferon genes and shows increased expression of interferon-regulated genes. We found that the resistance of H820 cells to influenza infection is likely linked to impaired viral entry—due to high basal levels of interferon-induced proteins known to inhibit endocytosis (IFITM1/2/3, NCOA7, and CH25H)—and to increased expression of mRNAs that encode other antiviral factors. In contrast, H322 cells show the absence or low levels of interferon-regulated genes involved in the inhibition of viral entry. These results suggest that the opposite phenotypes on viral entry of H322 and H820 cells may be at least in part associated with impaired or enhanced interferon response, respectively. Since most lung cancer patients have genomic characterization of their tumors, individualized differences in interferon responses may have therapeutic and patient management implications.

**IMPORTANCE:**

Lung cancers develop from genetic and epigenetic changes that can dramatically influence patients’ susceptibility to viral infection and replication. This study evaluates the responses to influenza virus infection of two patient-derived lung cancer cell lines. Interestingly, the cell lines investigated are of the same cancer type, lung adenocarcinomas, yet one cell line is highly susceptible, while the other cell line is highly resistant to viral infection. This is in part due to contrasting genetic alterations that lead to changes in the interferon response pathways, which differentially impact viral entry. Thus, identifying these risk factors can inform the prognosis of patients infected with influenza virus and guide their personalized treatment plans.

## INTRODUCTION

Lung cancer patients are predisposed to influenza virus infection, which can lead to hospitalization and increased risk of death (1). This highlights the importance of influenza vaccination in lung cancer patients as well as aggressive diagnosis and management of infection. It has been shown that lung cancer patients infected with influenza virus have difficulties clearing the virus due to immunosuppression (2). As a result of persistent infection, these patients develop chronic inflammation, which may increase the risk of tumor progression. In fact, repeated infection of cancer-free patients with influenza viruses has been associated with a high risk of developing lung cancer (3).

In addition to direct effects on the tumor, persistent viral infection can also alter the tumor microenvironment (TME) and promote or decrease tumor growth in response to cancer treatments (4). Activation of Toll-like receptors (TLRs) signaling generates proinflammatory cytokines that recruit immune cells to clear the virus. This pathway can contribute to pro-oncogenic TME and tumor cell survival (4). TLR7, for example, signals through interferon regulatory factors (IFRs), nuclear factor (NF)-κB, and mitogen-activated protein kinase (MAPK) pathways. All induce inflammation with the production of type I interferons (IFNs) and other cytokines. Chronic inflammation is associated with persistent viral infection and is known to inhibit cell death, recruit immunosuppressive cells, and promote tumorigenesis and metastasis (4). On the other hand, acute pro-inflammatory stimuli in lung tumors due to viral infection might change the immunosuppressive environment in the tumor, stimulating dendritic cell (DC) presentation and T cell priming, as well as NK and T cell killing of cancer cells (5).

Influenza virus infection can also induce both immunostimulatory or immunosuppressive effects that provide opposite effects on the TME for tumor growth, identifying a major knowledge gap. Namely, does influenza virus infection of patients with lung cancer increase or decrease their ability to mount an immune anti-tumor response? Prolonged viral infection leads to T cell inhibition and immunosuppression by upregulation of immunosuppressor ligands such as programed death ligand (PD-L1), PD-L2, indoleamine 2,3-dioxygenase 1, and galectin 9 (LGALS9) (6). For example, PDL-1 expression is stimulated during infection by IFN signaling via the signal transducer and activator of transcription 1 (STAT1) (7), potentially providing resistance to immune checkpoint blockade therapy. In fact, increased expression of interferon-stimulated genes (ISGs) is known to promote cell survival and resistance to radiation therapy and chemotherapy (6). Influenza virus-infected cancer cells can further evade immune cell detection by downregulating MHC-I expression, which also assists cancer cells to evade immune detection (8). This same change in the TME can also lead to resistance to cancer treatments by altering lipid and drug metabolism (6). On the other hand, viral infection of tumor cells can lead to cancer cell death and stimulation of anti-tumor immunity, and it is the basis of cancer virotherapy (9). Intratumoral administration of talimogene laherparepvec (T-VEC), a recombinant herpes virus, is among the current therapies for the treatment of melanoma, and several other viruses are under preclinical and clinical development for the treatment of tumors in combination with other anticancer therapies (10). Thus, respiratory virus infection in lung cancer patients might have different consequences, resulting in disparate outcomes such as exacerbated chronic viral disease and increased tumor progression or tumor regression due to acute immunostimulation. In addition, the permissiveness of lung cancer cells to viral infection likely influences the outcome of respiratory viral infection in a lung cancer patient.

In this study, we identified two patient-derived lung adenocarcinoma (LUAD) cell lines that have opposite responses to influenza virus infection and characterized potential molecular mechanisms involved in these differential responses. We found that reduced or enhanced interferon responses in these human lung cancer cells altered viral entry and viral gene expression due to genetic deletions of IFN genes or high copy number of these genes and of other genes involved in IFN response, respectively. These data emphasize the importance of assessing individual immunity to better determine the best treatment plans for both influenza virus infection and cancer.

## RESULTS

### Human lung cancer cell lines have differential susceptibility to viral infection

**Fig 1:**
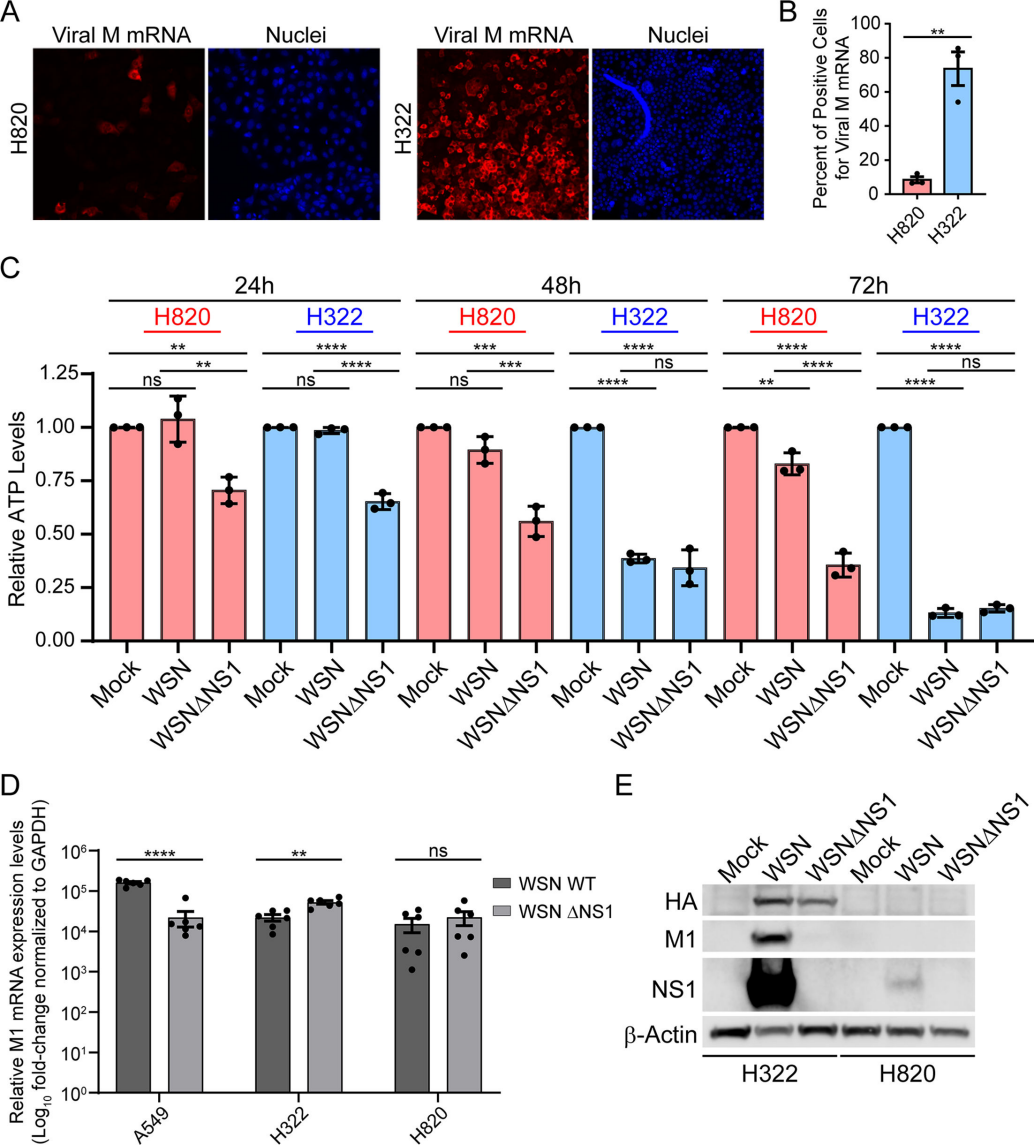
Human lung cancer cells H820 (resistant) and H322 (sensitive) show opposite responses to Influenza A virus infection. (A) smRNA-FISH images depicting H322 and H820 cell lines infected with influenza virus (A/WSN/33) at a multiplicity of infection (MOI) of 2 for 8 h. (B) Percent of cells stained for viral M mRNAs was determined by smRNA-FISH in the images shown in (A). (C) H820 or H322 cell lines were infected with wild-type influenza virus (A/WSN/33) or mutant A/WSN/33 ΔNS1 virus. Cells were infected at MOI of 0.1 for 24 h, 48 h, or 72 h. ATP levels were measured using Cell Titer Glo. (D) A549, H322, or H820 cells were infected with influenza virus at MOI of 1 for 16 h. Purified RNA from these cells was subjected to quantitative PCR (qPCR) to determine viral M mRNA levels, which were expressed relative to GAPDH levels in the respective cells. These conditions show similar levels of infection for each cell type—comparing A/WSN/33 vs A/WSN/33ΔNS1 viruses. (E) Cell extracts from H322 and H820 cell lines mock infected, infected with wild-type A/WSN/33, or infected with the mutant A/WSN/33ΔNS1 virus at MOI of 2 for 8 h were subjected to western blot analysis to detect the viral proteins HA, M1, and NS1. β-Actin is used as a loading control. Graphs show data points and mean ± SD (B and C) or mean ± SEM (D). ns, not significant, **P < 0.01; ***P < 0.001; ****P < 0.0001. In panels (B and D) *P* values were calculated using unpaired two-tailed Student’s *t* test. In panel (C), *P* values were calculated using Tukey’s (one-way analysis of variance [ANOVA]) multiple comparisons.

To evaluate the resistance of various human lung cancer cell lines to influenza A virus (A/WSN/33) infection, we used single-molecule RNA fluorescence *in situ* hybridization (smRNA-FISH) to detect viral M mRNA in cancer cells derived from lung tumors after viral exposure. We identified two LUAD cell lines (NCI-H820 and NCI-H322) with opposite phenotypes—H820 shows resistance to influenza A viral infection, while H322 is highly susceptible to influenza infection (Fig. 1A and B). Since viral M mRNA levels were analyzed 8 h post-infection (hpi), within a single viral life cycle, a decrease in percent infection can be due to a defect in viral entry, trafficking into the nucleus, and/or a decrease in mRNA biogenesis or processing. In contrast, high susceptibility to infection could be due to an enhancement of any of these processes.

These observed phenotypes were also assessed by monitoring cell viability at 24 h, 48 h, and 72 h post-infection with influenza virus (A/WSN/33; Fig. 1C). In addition, both cell lines were infected with A/WSN/33 mutant lacking the NS1 virulence factor (WSNΔNS1). The pathways that NS1 uses to promote pathogenesis are well studied, and differences between these two infection conditions (A/WSN/33 vs A/WSN/33 ΔNS1) can provide insights into the mechanisms underlying the susceptibility or resistance of these cell lines to influenza infection. In general, WSNΔNS1 induces more cell death than the wild-type virus due to the loss of anti-apoptotic effects of NS1 (11). Additionally, WSNΔNS1 effectively replicates in cells or animals with interferon-deficient response, as NS1 promotes viral infection by inhibiting the type I IFN system (12) and expression of host mRNAs that encode antiviral factors (13). The H322 cell line showed a significant decrease in cell viability 24 h post-infection with WSNΔNS1 compared to the wild-type virus (Fig. 1C). H322 cell viability was further decreased at 48 h and 72 h in both WSN and WSNΔNS1 infected cells relative to uninfected controls. In contrast, H820 cells showed a much slower decline in cell viability compared to H322 cells at 48 h and 72 h post-infection with both viruses, indicating resistance to infection compared to H322 cells. Nevertheless, WSNΔNS1 infection resulted in more cell death than WSN infection in H820 cells. In contrast, H322 cell death was higher than in H820 cells both in the presence and absence of NS1, suggesting faster infection leading to cell death. Each cell line was equally infected by wild-type WSN and WSNΔNS1 as determined by qPCR of viral M1 mRNA (Fig. 1D).

To further assess the differences in susceptibility to influenza virus infection between these cell lines, we determined viral protein levels (HA, M1, and NS1) by western blot analysis in both H322 and H820 cells infected with WSN or WSNΔNS1 (Fig. 1E). Consistent with the experiments determining percent infection, H322 cells expressed more viral proteins than H820 cells. When infected with WSNΔNS1, the HA viral protein was detected in H322 cells and not in H820 cells, while neither HA nor M1 proteins were detected in H820 cells, likely due to the lack of the viral NS1 protein, which promotes nuclear export of HA and M1 mRNAs for translation into their respective proteins in the cytoplasm (14, 15). Together, these results indicate that optimal expression of viral proteins requires NS1 expression in H322 cells, while little viral protein expression is achieved for both WSN and WSNΔNS1 in H820 cells.

**Fig 2:**
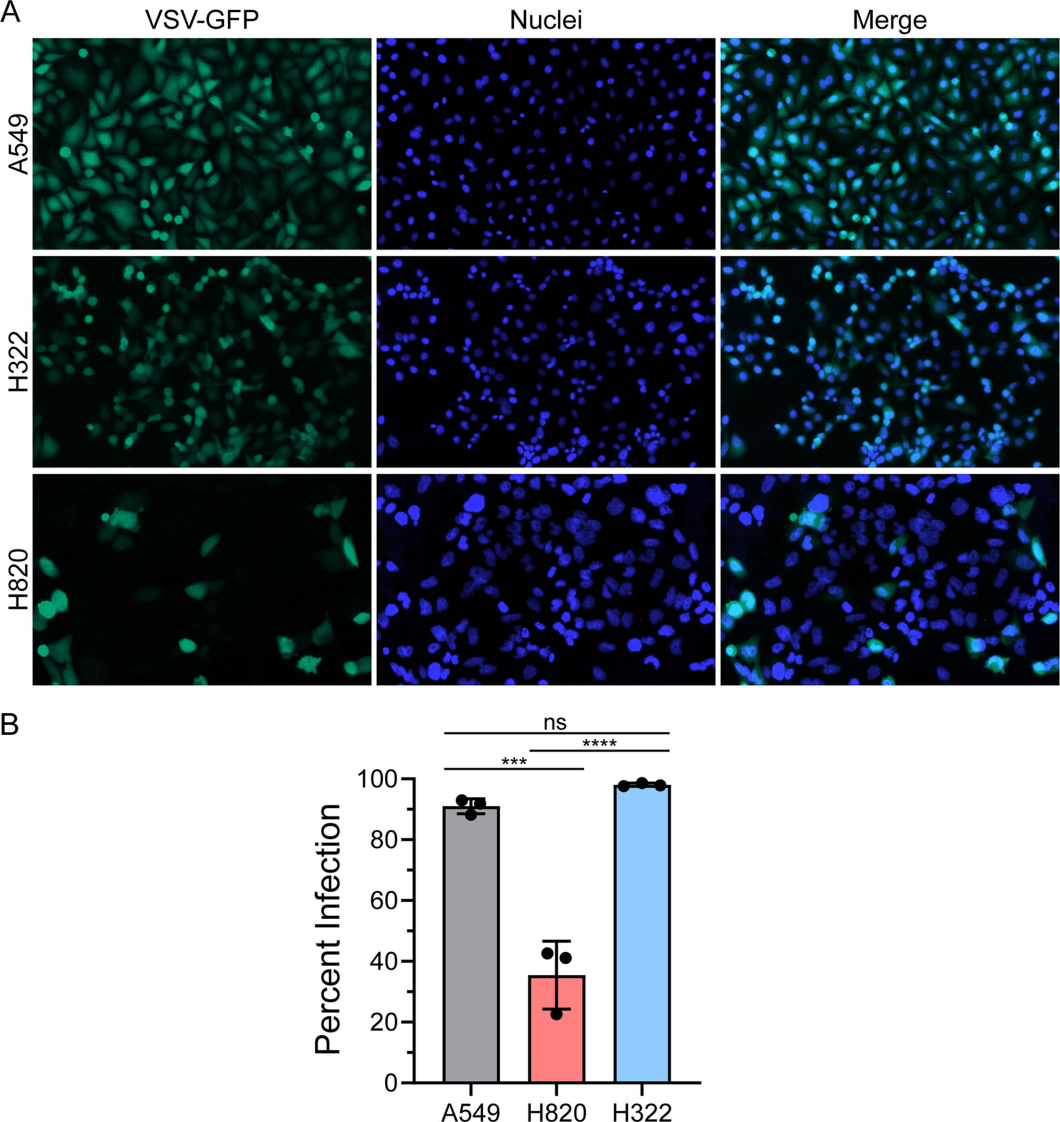
H820 cells are resistant to VSV infection while H322 cells are sensitive. (A) Immunofluorescence microscopy detected GFP protein in A549, H322, and H820 cell lines infected with VSV-GFP at a multiplicity of infection (MOI) of 0.1 for 8 h. (B) Percent infection was determined by the number of cells positive for GFP in (A). The Graph shows data points and mean ± SD. ns, not significant; ***P < 0.001; ****P < 0.0001. *P* values were calculated using Tukey’s (one-way ANOVA) multiple comparisons.

To determine whether these differences in viral susceptibility are specific to influenza virus, we infected these cells with vesicular stomatitis virus (VSV), another RNA virus sensitive to the IFN system. A549, H322, and H820 cells were infected with VSV expressing GFP, and percent infection was determined. Immunofluorescence images show increased VSV-GFP-positive cells in both A549 and H322 compared to H820 cells (Fig. 2A), which is similar to what we observed with the increase in influenza virus infection in H322 compared to H820 cells (Fig. 1A and B). Image quantification further demonstrates these effects and reveals a slight but statistically significant increase in VSV-GFP signal in H322 cells compared to A549 cells (Fig. 2B). Together, these results show that infection of H322 and H820 cells with two diverse RNA viruses had similar effects in their susceptibility or resistance to infection, respectively, suggesting that there is disruption of a pathway(s) that affect replication of both viruses. Among the well-known pathways commonly impacting both viruses is the interferon pathway, which could provide resistance against infection in H820 cells and could be defective in H332 cells, resulting in its increased susceptibility to infection.

#### The cell line susceptible to viral infection (H322) has deletions in IFN genes, while the resistant cell line (H820) has a high copy number of the same IFN genes and of other genes involved in IFN response.

**Fig 3:**
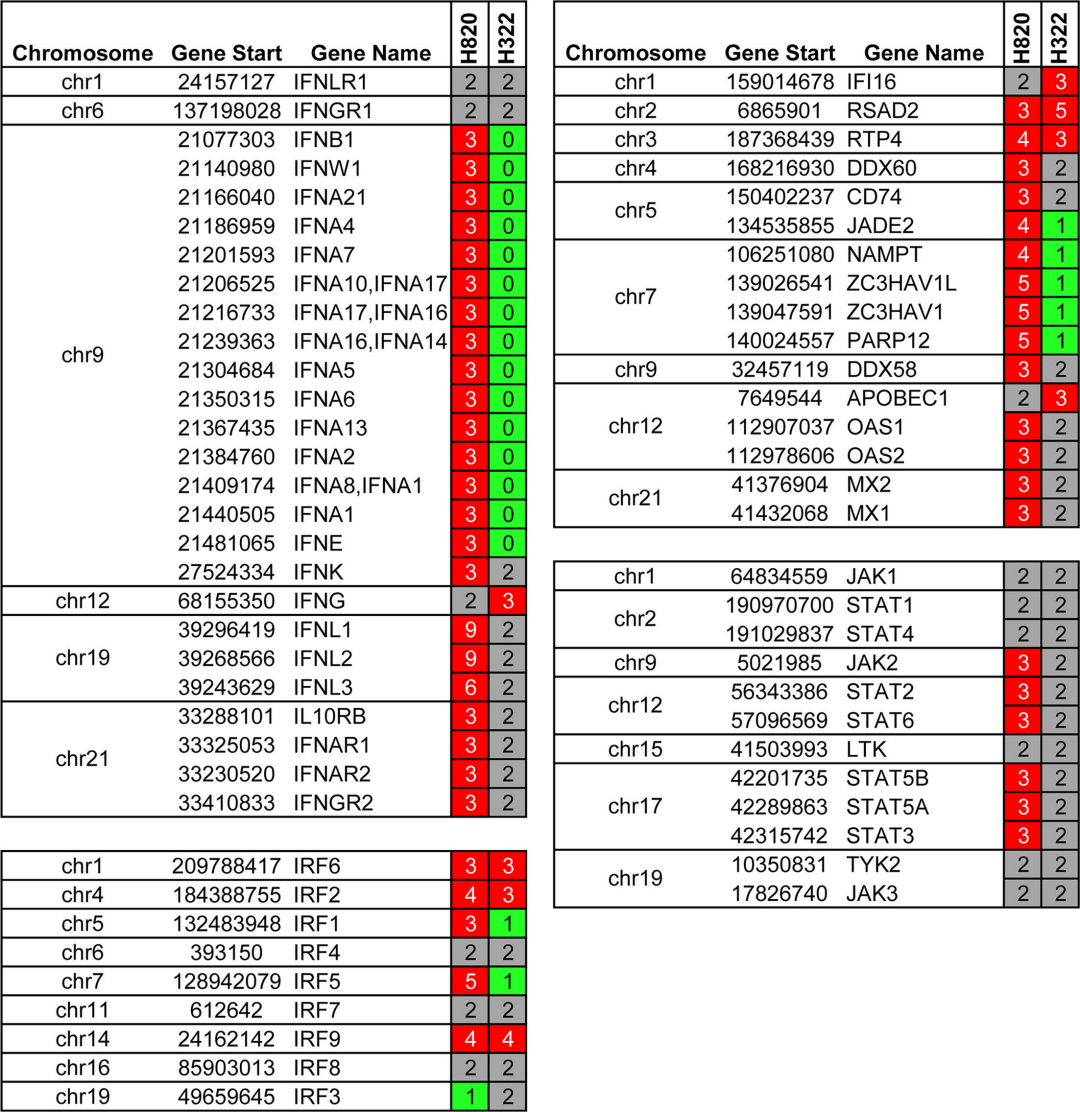
Influenza A virus and VSV susceptible cell line (H322) have the deletion of IFN genes, while the resistance cell line (H820) has an amplification of IFN genes and other genes in the IFN pathway. The table shows copy number variation (CNV) data of the indicated chromosomes from H322 and H820 cell lines. The table shows gene deletions highlighted in green and amplifications highlighted in red.

We then analyzed the genomic sequence of both H322 and H820 cells. Interestingly, the H322 cells have a deletion of a region in both copies of chromosome 9 that includes *IFNB1*, *IFNW1*, *IFNE*, and several *IFNA* genes (Fig. 3), while other IFN genes (*IFNL1*, *IFNL2*, *IFNL3*, and *IFNK*) have two copies, *IFNG* has three copies, and interferon receptors have both gene copies. Additionally, H322 has a single copy number of the genes encoding the interferon response factors IRF1 and IRF5 as well as of several ISGs, while IRF2, IRF6, and IRF9 have high copy numbers. Other IRF genes and IFN receptor genes are present in at least two copies (Fig. 3). Thus, the deletion of various IFN genes and single copy number of genes involved in IFN response may contribute to the observed susceptibility to influenza virus and VSV infection despite the high copy number of some IRF genes. In contrast, H820 cells show a duplication in the same chromosome 9 region, resulting in three copies of these same interferon genes described above (*IFNB1*, *IFNW1*, *IFNE*, and several *IFNA* genes) as well as nine copies of *IFNL1* and *IFNL2*, and six copies of *IFNL3*. In addition, these cells show high numbers of *IRF1*, *IRF2*, *IRF2BP2*, *IRF5*, *IRF6*, and *IRF9* genes and a single copy of the *IRF3* gene (Fig. 3). H820 cells also have three copies of the JAK2 and STAT2 genes and of ISGs including OAS1, OAS2, MX1, MX2, and others listed in Fig. 3. The striking number of genes with high copy numbers, including IFNs and constituents of the IFN response pathways, suggests that the IFN response may be robust in the H820 cells and potentially contributes to the observed resistance to viral infection.

### Immune pathways are differentially regulated in human lung cancer cells

**Fig 4:**
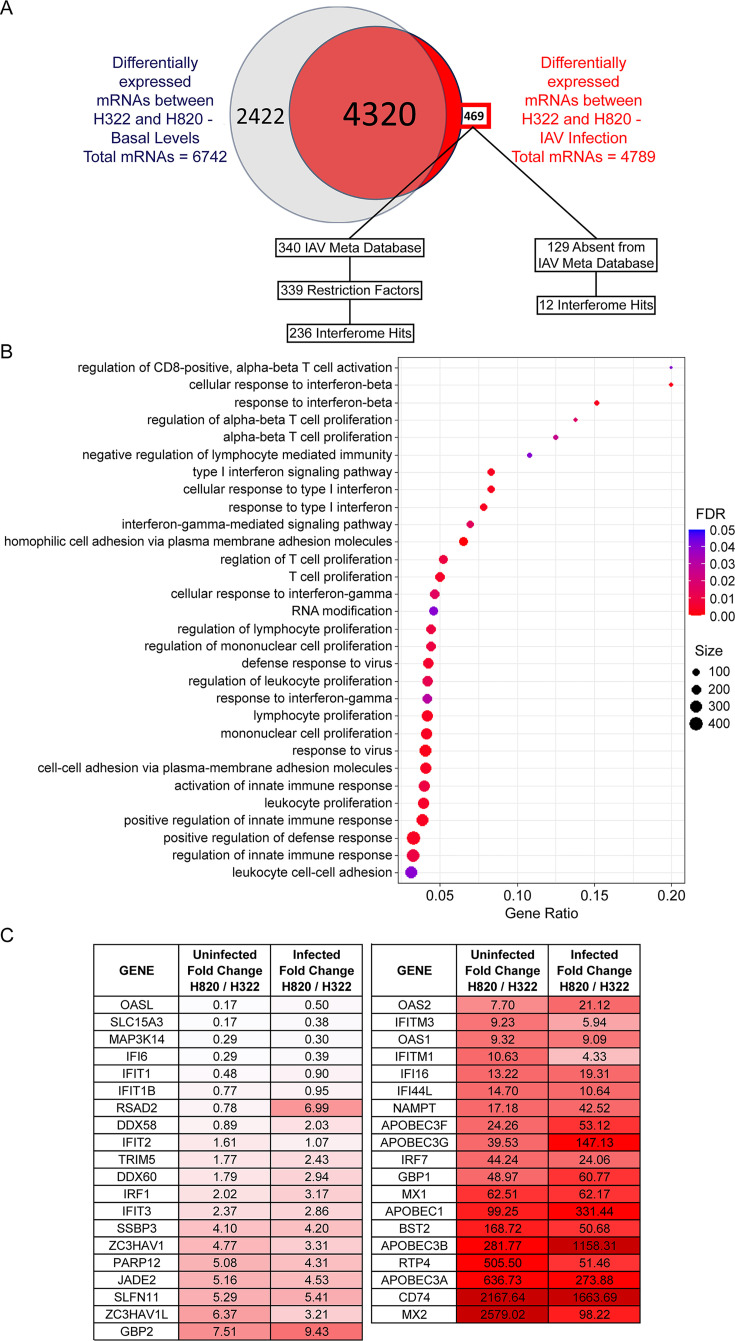
RNAseq analysis of H322 and H820 cells in the absence of infection or upon infection with influenza virus. (A) Venn diagram shows the number of mRNAs that are differentially expressed between H820 and H322 cells at basal levels or upon infection with influenza virus at a multiplicity of infection (MOI) of 1 for 8 h. These include mRNAs that are exclusively expressed in either one of these cell lines. Hits that were differentially expressed exclusively during infection were further compared to the Interferome and IAV Meta Database. (B) Graph of ORA for RNAs upregulated in H820 cells compared to H322 cells upon infection with A/WSN/33 (WSN) at MOI of 1 for 8 h. Each dot represents a pathway that is upregulated in H820 cells, where the size of the dot indicates the number of RNAs that contribute to that pathway, and the color represents the false discovery rate (FDR). Gene ratio represents the Log2-fold change difference between H820 over H322 cells. (C) Examples of ISGs differentially expressed in H820 cells compared to H322 cells in non-infected and/or infected conditions. Fold change between these cell lines is shown.

To further investigate the cellular pathways differentially regulated in both uninfected and infected H322 and H820 cells, we performed RNAseq analysis under these various conditions. We found that most of the pathways upregulated in H322 cells upon infection are related to the immune response to viral infection. In addition, interferon-related pathways were also upregulated, likely due to interferon λ production and type III interferon responses. However, the induction of these pathways in H322 cells was not sufficient to provide protection against influenza virus infection and replication. Additionally, RNAseq analysis of H820 cells uninfected or infected with influenza virus showed that many immune response pathways were also upregulated in H820 cells upon infection. In influenza virus-infected cells, 4,789 mRNAs were differentially expressed between both H322 and H820 cell lines, though most of these mRNAs were already differentially expressed in uninfected conditions (Fig. 4A). Interestingly, only 469 mRNAs were differentially expressed exclusively in infected conditions (Fig. 4A; Table S1, Tab 1). These 469 mRNAs are of particular interest, as they may encode antiviral factors. Thus, we compared these 469 mRNAs with both the Interferome (16) and Influenza A Virus (IAV) Meta Database (17). The latter includes resistors and sensitizers of influenza virus infection. Indeed, we found that out of these 469 mRNAs (Table S1, Tab 1), 340 mRNAs are present in the IAV Meta Database (Table S1, Tab 2), among which are 339 mRNAs that encode restriction factors (Table S1, Tab 3), and 236 of them are interferon regulated (Table S1, Tab 4; Fig. 4A). Additionally, out of the 469 mRNAs, 129 mRNAs were not present in the IAV Meta Database, but 12 of these mRNAs are interferon regulated (Table S1, Tab 5; Fig. 4A). This left 117 mRNAs that are excluded from both databases, which are possible novel regulators of viral infection (Fig. 4A; Table S1, Tab 5). Next, overrepresentation analysis (ORA) showed that the majority of the pathways upregulated in H820 cells among the 469 mRNAs are immune related (Fig. 4B). These include interferon response to infection and immune cell signaling involving several T cell regulatory pathways. Constitutive levels of IRF-1, ISGs, and other immune factors are detected in H820 cells (Fig. 4C; Table S1), and their expression levels are further increased upon infection. This is not the case for most of these mRNAs in H322 cells. Moreover, low basal levels of IFNα1, IFN λ1, and IFNεmRNAs are detected in H820 cells and not in H322 cells (Table S1, Tab 6). Thus, differential expression levels of these immune factors likely contribute to the contrasting antiviral response observed between H820 and H322 cells.

### LUAD cells susceptible to viral infection have deficient interferon responses, while LUAD cells resistant to infection have robust interferon responses

**Fig 5:**
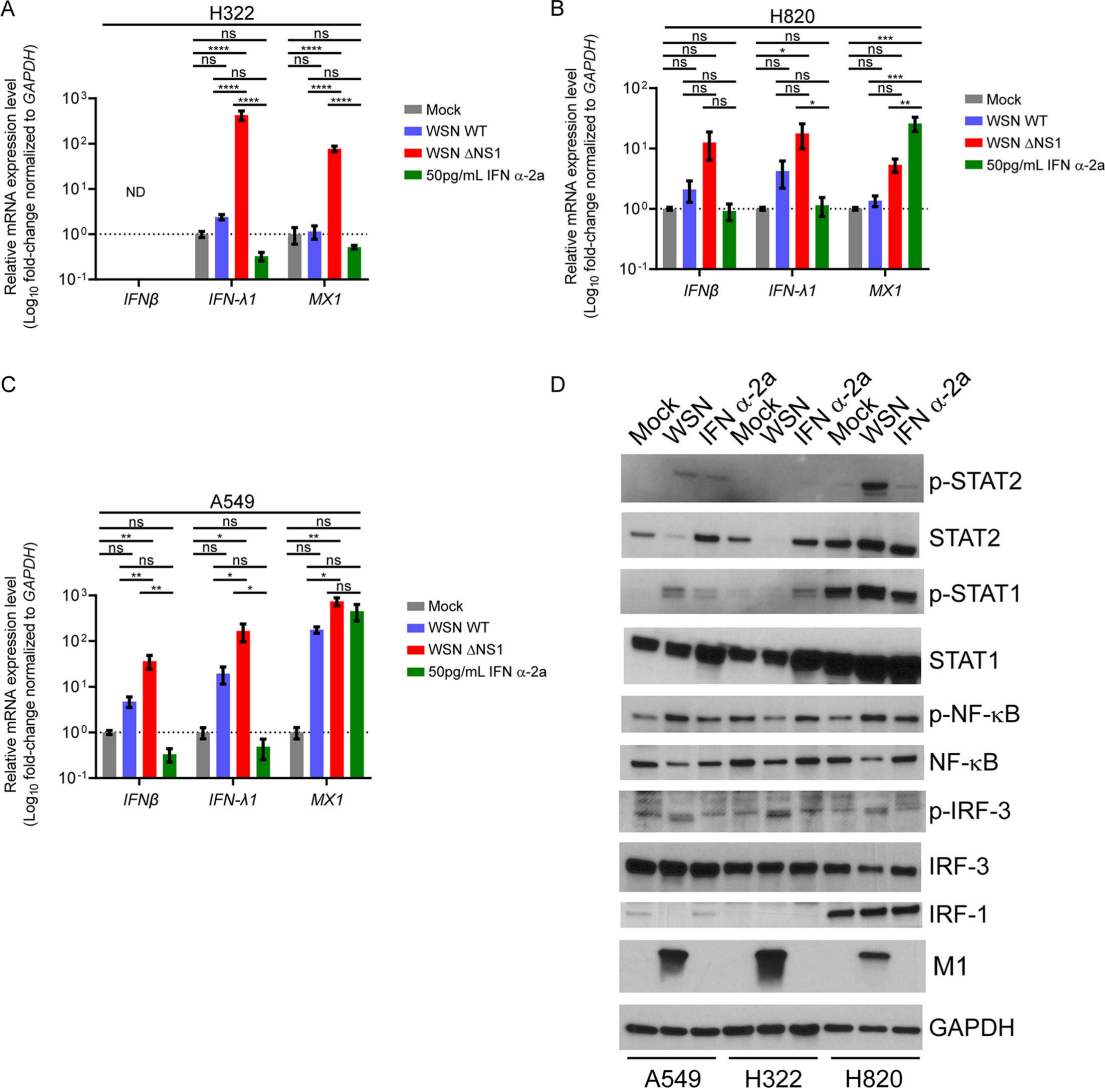
H322 cells do not upregulate IFNα and β genes upon viral infection, while H820 cells induce IFN. H322 (A), H820 (B), or A549 (C) cells were infected with A/WSN/33 or A/WSN/33ΔNS1 at a multiplicity of infection (MOI) of 1 for 18 h or treated with IFNα-2a for 18 h. RNA was purified from each cell line and subjected to qPCR to determine the levels of *IFNβ*, *IFN-λ1*, and *MX1* mRNAs. Bars represent mRNA levels relative to GAPDH. (D) Cells were either mock-infected and mock-treated, infected with A/WSN/33 at an MOI of 1, or treated with 50 pg/mL of IFN α-2a and then subjected to western blot analysis to detect p-STAT2, STAT2, p-STAT1, STAT1, p-NF-κB, NF-κB, p-IRF-3, IRF-3, IRF-1, and M1 proteins. GAPDH is used as a loading control. Graphs show means ± SEM. ND = not detected, ns = not significant, *P < 0.05; **P < 0.01; ***P < 0.001; ****P < 0.0001. *P* values were calculated using Tukey’s (one-way ANOVA) multiple comparisons.

Since H322 cells have several type I IFN genes deleted, we next tested the transcriptional response with the addition of exogenous interferon. H322, H820, and A549 cells were infected with wild-type WSN, WSNΔNS1, or treated overnight with 50 pg/mL IFNα-2a. RNA purified from these cell lines was subjected to real-time RT-PCR to measure IFNβ, IFNλ1, and MX1 (an ISG) mRNA levels. Consistent with the copy number variation (CNV) data, we did not detect IFNβ mRNA in H322 cells due to gene deletion (Fig. 5A). In H322 cells, MX1 mRNA levels increased when infected with WSNΔNS1, which is likely mediated by IFNλ as IFNλ genes are present in these cells. IFNλ1 and MX1 levels were downregulated upon IFNα-2a treatment compared to mock conditions, and only a slight increase in IFNλ1 and no change in MX1 expression were observed upon infection with the wild-type virus (Fig. 5A). Thus, treatment with IFNα-2a was not sufficient to upregulate MX1 mRNA levels, indicating additional defects in the IFNα-2a response pathway.

In H820 cells, IFNλ1 and MX1 mRNA levels were upregulated during infection with WSNΔNS1, and only a slight increase in IFNλ1 was observed in wild-type WSN-infected cells (Fig. 5B). Of note, the efficiency of infection is low compared to H322 cells. In addition, treatment with IFNα-2a upregulated MX1 mRNA levels with no change in IFNλ1 mRNA levels. A549 cells were tested as a positive control (Fig. 5C). These cells showed the expected increase in IFNλ1 and MX1 mRNA levels under both infection conditions and no change in IFNλ1 mRNA levels upon IFNα-2a treatment. Both H820 (Fig. 5B) and A549 (Fig. 5C) show increased IFNβ expression levels upon infection, with a greater increase observed upon WSNΔNS1 infection. Moreover, treatment with IFNα-2a resulted in no change in IFNβ levels. Overall, H820 cells induced an interferon response as opposed to the impaired antiviral response shown in H322 cells. Infection with WSNΔNS1 resulted in the largest fold increase in most mRNAs tested in all three cell lines. Since one of the several functions of the NS1 virulence factor is to inhibit interferon responses, cellular antiviral response is best induced under infection conditions in the absence of NS1.

In addition to mRNA levels, protein levels of several immune-related genes were increased in H820 cells in basal, infection, and interferon-treated conditions (Fig. 5D). Upon infection with A/WSN/33 or interferon treatment, A549 cells show increased phosphorylation of STAT1, STAT2, and NF-κB. Infection of H322 cells with A/WSN/33, however, reduced phosphorylation of NF-κB in these cells. Furthermore, phosphorylation of STAT1 in H322 cells was observed only under IFN treatment, while STAT2 phosphorylation was not detected in these cells upon viral or IFN treatment. Of note, STAT2 levels decreased upon infection of H322 cells. Regarding H820 cells, STAT1 and NF-κB were phosphorylated in basal conditions, during infection, and upon IFN treatment, although there was an increase in their levels during infection. STAT2 was phosphorylated only under infection conditions in H820 cells. Importantly, IRF-1 was abundantly present under all conditions in H820 cells. High basal levels of IRF-1 in H820 cells likely lead to a robust immune response that contributes to the strong antiviral phenotype observed in this cell line. This effect probably induces high mRNA levels of ISGs listed in Fig. 4C, including MX1 and OAS2.

**Fig 6:**
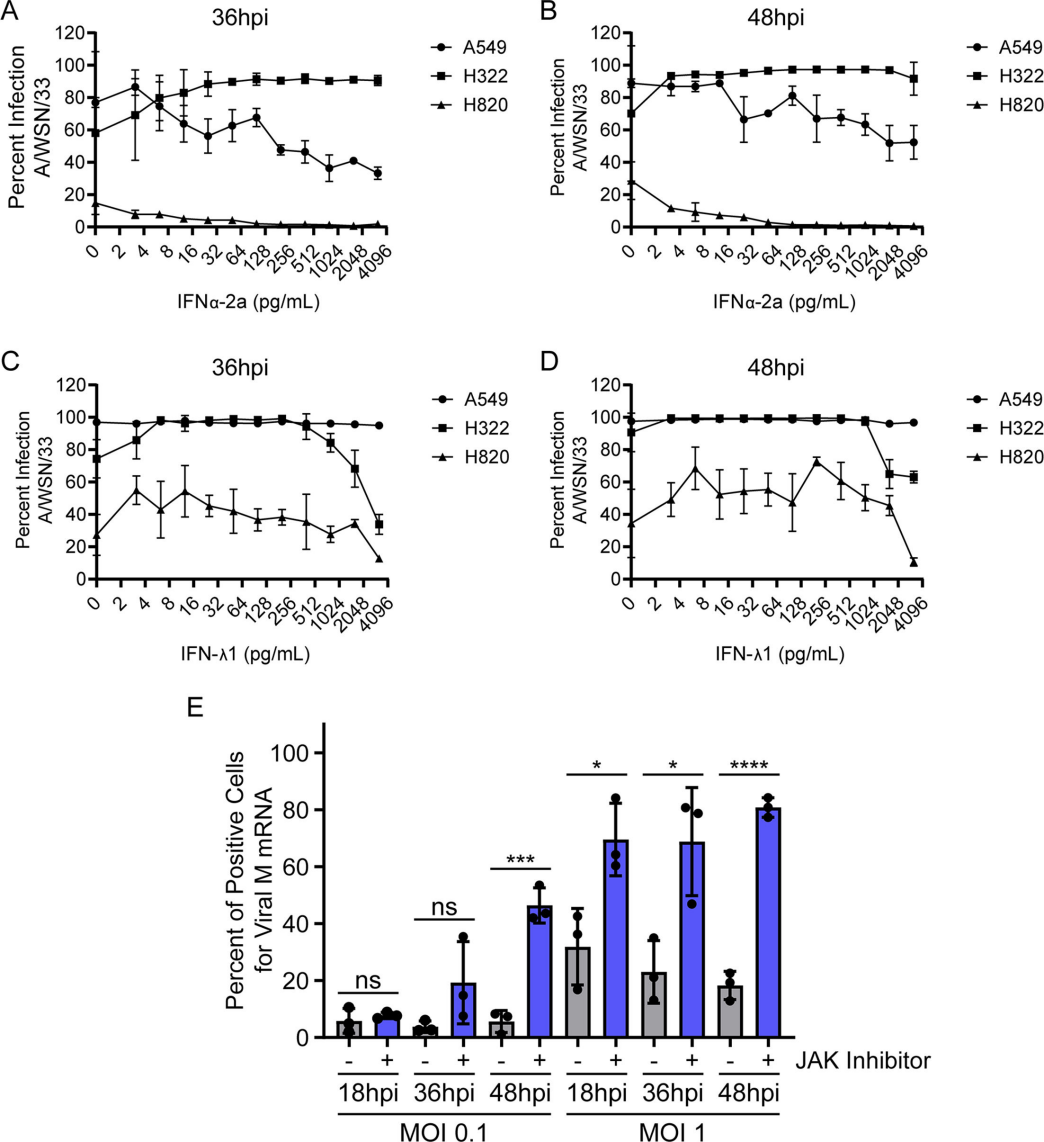
Interferon response is lacking in the highly IAV-susceptible cell line (H322), while the resistant H820 cell line remains responsive. (A and B) A549, H322, and H820 cells were infected with A/WSN/33 at 0.1 multiplicity of infection (MOI), and percent infection was determined in the presence of increasing concentrations of human interferon-α2a at 36 hpi (A) or 48 hpi (B). Interferon treatment started at the time of infection. (C and D) A549, H322, and H820 cells were infected with A/WSN/33 at 0.1 MOI, and percent infection was determined in the presence of increasing concentrations of human interferon-λ1 at 36 hpi (C) or 48 hpi (D). Interferon treatment started at the time of infection. Graphs show mean ± SD. (E) H820 cells were treated with dimethyl sulfoxide (DMSO) (control) or with 10 µM ruxolitinib (JAK inhibitor) for 1 h and then infected with A/WSN/33 for 18 h, 36 h, and 48 h followed by smRNA-FISH to detect viral M mRNA. The percentage of positive cells for M mRNA was determined. Data points, mean, and SD are shown. ns = not significant, *P < 0.05, ***P < 0.001, and ****P < 0.0001. *P* values were calculated using unpaired two-tailed Student’s *t* test.

We next determined the impact of interferon treatment on influenza virus infection over time in these cell lines. We performed dose-response curves with IFNα-2a and IFN-λ1 and measured percent infection in H322, H820, and A549 cells (Fig. 6). Upon treatment with IFNα-2a, there was a dose-dependent decrease in the percentage of infected A549 cells at both 36 h and 48 h post-infection (Fig. 6A and B). H820 cells showed low infection levels, which further decreased at high levels of interferon treatment (Fig. 6A and B). Regarding H322 cells, there was no decrease in percent infection at either timepoints, maintaining a high infection rate at all IFNα-2a concentrations tested (Fig. 6A and B). Thus, in addition to several types of interferons missing in H322 cells, these cells have an aberrant response to IFN α-2a. On the other hand, the percent infection decreased in both H322 and H820 cells at high concentrations of IFN-λ1 (Fig. 6C and D), as both cells have intact IFN-λ1 response. Infection of A549 cells was not affected by increasing concentrations of IFN-λ1 in the range tested (Fig. 6C and D); however, at higher concentrations of IFN-λ1 (1,000 ng/mL), viral infection was reduced by ~20% in A549 cells (Fig. 1). This modest decrease in infection has also been shown by others (18), where 500 ng/mL of IFN-λ decreased infection by ~30% in A549 cells. This may reflect low expression levels of IFN-λ1 receptor in these cells.

To further determine the impact of IFN signaling on the resistance of H820 cells to viral infection, we treated these cells with a non-cytotoxic concentration of ruxolitinib (JAK and Tyk2 inhibitor) and infected them with influenza A virus. As shown in Fig. 6E, JAK and Tyk2 inhibition increased H820 susceptibility to influenza virus infection. Fig. S2 shows that the concentration of ruxolitinib used in these conditions does not decrease cell viability. In sum, these results indicate that H820 cells have a robust IFN response that likely contributes to their resistance to viral infection.

### Viral entry is impaired in the viral-resistant cell line, while the viral-susceptible cells show robust entry

**Fig 7:**
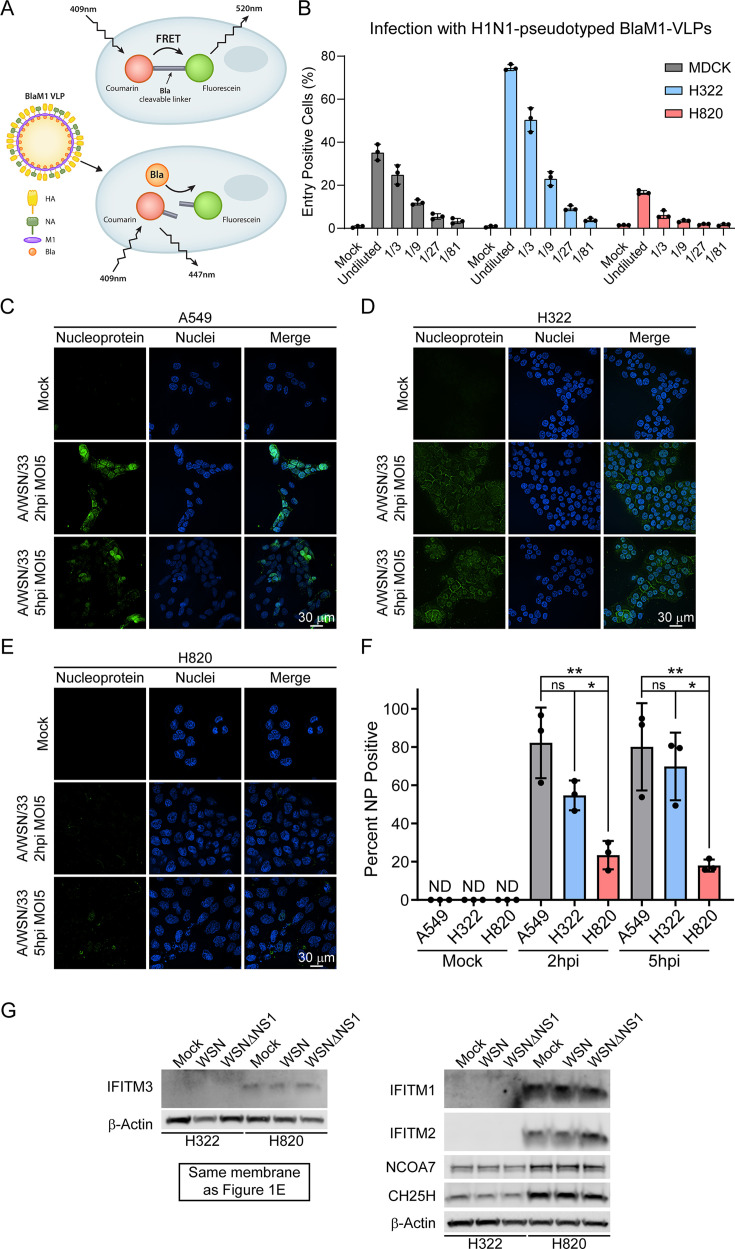
Influenza virus entry is impaired in cell line resistant to viral infection (H820) and not in the highly susceptible cell line (H322). (A) Diagram of BlaM1-VLPs entry assay. VLPs consist of viral proteins HA, NA, and M1 from the A/WSN/33 viral strain, with M1 fused to beta-lactamase (Bla) protein. Cells are incubated with the BlaM1-VLPs, then with a fluorescent substrate (CCF2-AM) consisting of Coumarin and Fluorescein linked by a linker cleavable by Bla. In cells that were not infected with the BlaM1-VLP, excitation at 409 nm results in fluorescence resonance energy transfer (FRET) occurring between Coumarin and Fluorescein, resulting in Fluorescein emitting at 520 nm. In cells infected with the BlaM1-VLP, Bla is present to cleave the Coumarin and Fluorescein. When excitation occurs at 409 nm, there is no FRET, and only Coumarin emits at 447 nm. This difference in excitation is used to determine if cells are positive for BlaM1-VLP entry. (B) MDCK, H322, or H820 cells were incubated with BlaM1-VLPs, the substrate was added, and Bla activity was measured by flow cytometry. Various VLP dilutions were used as indicated in the x-axis. Each bar represents the percent positive cells from 10,000 live cells. Data represent three independent experiments. (C–E) Representative images of synchronized viral entry in A549 (C), H322 (D), and H820 (E) cell lines infected with A/WSN/33 at a multiplicity of infection (MOI) of 5 after 2 hpi or 5 hpi. NP protein (green) present in nuclei (blue) was counted as positive for viral entry. (F) Graphs show the percentage of NP-positive cells. Bars represent means ± SD, and each point represents the percentage of a single replicate, *n = 3*. ns, not significant; *P < 0.05; **P < 0.01. *P* values were calculated using Tukey’s (one-way ANOVA) multiple comparisons. (G) Cell extracts from H322 and H820 cells were mock infected, infected with wild-type A/WSN/33, or infected with A/WSN/33 ΔNS1 at MOI of 2 for 8 h and then subjected to western blot analysis to detect IFITM1/2/3, NCOA7, and CH25H proteins. Cell lysates are the same as in Fig. 1E. IFITM3 and β-Actin were probed in the same membrane shown in Fig. 1E. The other proteins were probed using a different membrane. *n* = 3. β-Actin is used as a loading control.

Since H820 cells have a high copy number of IFN genes and show a low infection rate, we next sought to determine whether this mechanism of resistance involves viral entry. To this end, we incubated H820 cells, H322 cells, or MDCK (control) cells with viral-like particles (VLPs) carrying a fusion protein of a beta-lactamase reporter protein (Bla) and influenza matrix protein-1 (M1), referred to as BlaM1-VLPs (Fig. 7A). These BlaM1-VLPs mimic WSN viral entry, and cells positive for entry are determined by detecting BLA activity using flow cytometry (19). Using a serial dilution of BlaM1-VLPs, we detected a gradual decrease in the percentage of positive cells in MDCK cells with progressively lower concentrations of VLPs (Fig. 7B). Relative to MDCK cells, H322 has an increase in positive cells upon incubation with the VLPs (Fig. 7B). However, H820 cells showed a decrease in the percentage of positive cells compared to MDCK and H322 cells (Fig. 7B). This result is supported by synchronized infection of A549 (used as a reference), H322, and H820 cells with A/WSN/33 followed by early detection of the viral nucleoprotein (NP) to assess viral entry. Cells were treated with cycloheximide to ensure that the NP detected was derived from the virus during the initial infection and not from a newly translated protein. After 2 h and 5 h post-infection, cells were fixed, and immunofluorescence was performed to detect NP. A549 cells were used as a reference and, together with H322 cells, showed a higher percentage of NP-positive cells than H820 cells (Fig. 7C through F). These data indicate that viral entry is enhanced in H322 cells, and there is a potential viral entry defect in H820 cells. Therefore, we then looked at the levels of interferon-induced proteins that have been shown to inhibit viral entry (Fig. 7G). These include IFITM1, IFITM2, IFITM3, CH25H, and NCOA7, which regulate the endocytic pathway (20). IFITM3 protein was not detected in H322 cells but is present in H820 cells (Fig. 7G). In addition, IFITM1, IFITM2, CH25H, and NCOA7 are present in higher levels in H820 cells than in H322 cells, further documenting the robust interferon response of H820 cells. Thus, the increased levels of these factors involved in the inhibition of viral entry may contribute to the reduced endocytosis of the BlaM1-VLPs in H820 cells and to the observed resistance to influenza virus and VSV infections. Taken together, abnormal regulation of viral entry and expression of other genes involved in the IFN pathway likely contribute to the contrasting phenotypes of these human lung cancer cells.

## DISCUSSION

Early in cancer development, tumors exhibit features that differentiate them from normal cells, alert immune cells, and allow tumors to be recognized and eliminated by the immune system. In patients who develop clinically evident cancer, tumors are able to bypass these mechanisms due to genetic and epigenetic changes that suppress the immune system, permitting tumor cells to progress as part of the “hallmarks of cancer” (21). Recent efforts have resulted in treatments that block the immune checkpoint pathways and release immunosuppression, leading to durable clinical anti-tumor responses in a subset of cancer patients (22, 23). These findings suggest that targeting the mechanisms tumor cells utilize to evade the host immune system may lead to more effective immune-modulating treatments. One of our reasons for studying the ability of influenza virus to infect and replicate in lung cancers is to determine if tumor cell features related to deficits in tumor cell immune signaling detected by virus replication may ultimately yield insights into how tumors evade immune responses. In the current study, we identified dramatic differences in LUAD susceptibility to influenza virus infection that are related to intra-tumor cell immune signaling pathways that differ because of tumor-acquired genetic changes.

We identified two patient-derived LUAD cell lines that are markedly different in their ability to support influenza virus replication. LUAD H322 cells demonstrated increased susceptibility to viral infection, while LUAD H820 cells were resistant to infection. Consequently, H322 cells showed increased cell death upon infection with influenza virus compared to H820 cells. This increased susceptibility to death upon infection of H322 cells was similar to when they were infected with a mutant influenza virus lacking the virulence factor NS1. This result suggested a potential deficiency in interferon expression or response in H322 cells, as NS1 is known to inhibit the type I IFN response (13). This was then confirmed by chromosomal CNV analysis, which showed a deletion of both copies of several IFN genes in H322 cells, while there were three copies of these same IFN genes in H820 cells. Additionally, H820 has increased copy numbers of several ISGs and other genes that play a role in immune-related pathways, including MX1, MX2, OAS1, OAS2, JAK2, and STAT2. In fact, H820 cells have high relative mRNA levels of several ISGs compared to the susceptible H322 cell line. Of note, H820 cells express high levels of IRF1 protein, which induces several ISGs, and of STAT1 protein. Treatment of H820 cells with a JAK inhibitor increased the susceptibility of these cells to infection, demonstrating the importance of JAK/STAT signaling in the resistance of H820 cells to viral infection. Thus, analysis of IFN responses in H322 and H820 cell lines indicates that their genetic abnormalities and aberrant expression of proteins involved in the IFN pathway appear to contribute to their opposing responses to influenza virus infection.

H322 cells have an unresponsive IFN pathway, while H820 cells express high levels of ISGs that likely contribute to the observed decrease in viral entry. Among the ISGs expressed in high levels in H820 cells are IFITM1/2/3, CH25H, and NCOA7, which are known IFN-inducible proteins with antiviral activities. IFITM proteins have been shown to alter the properties of the plasma membrane to prevent fusion of the viral membrane with the cellular membrane (24–27). CH25H alters components of cellular membranes by converting cholesterol to 25-hydroxycholesterol (25HC) (28). The latter has been found to broadly inhibit the entry of various enveloped viruses, and 25HC-deficient mice are susceptible to viral infection (29). NCOA7 interacts with the vacuolar H^+^-ATPase (V-ATPase) to induce endosomal acidification, lysosomal protease activity, and antigen degradation during viral entry. NCOA7 inhibits the fusion of the influenza virus membrane with the endosomal membrane (30). Since H820 has robust interferon signaling and high levels of these ISGs, the cellular membrane is likely primed to prevent viral membrane fusion, which is a contributing factor for providing robust resistance to influenza virus infection.

The importance of interferon in the immune response to viral infection has been well studied. Mice without type I IFNRs or STAT1 signaling are susceptible to influenza virus infection (6, 31) and other viruses (31–34). Since non-tumor cells typically have a normal interferon response while various tumor cells can have deficits in interferon responses, it has been shown that viruses such as VSV preferentially replicate and kill these immune-deficient tumor cells. This resulting viral-mediated oncolytic effect has made this phenotype the basis for developing cancer treatments by utilizing viruses or viral vectors (35). In fact, an example of a safety method for developing these therapies is to include the human *IFNβ* gene in the VSV genome to protect normal cells (36, 37). While this treatment may selectively infect and produce an oncolytic effect, other cancer cells such as H820, with robust interferon response, would likely be non-responsive to this treatment. Another key observation regarding the susceptible H322 cell line, which lacks several type I interferon genes typically associated with immune response to viral infection, is that these cells showed intact type III interferon expression that triggered an insufficient innate antiviral immune response to influenza virus. While these pathways were not sufficient to provide resistance to influenza infection, it is possible that they are able to provide resistance against other respiratory viruses. In SARS-CoV-2 infection, for example, injection of interferon λ in infected patients results in faster clearance of the virus than placebo control (38). As personalized medicine becomes more prevalent and accessible, the need for identifying key factors for determining the best health care for lung cancer patients also grows. While research on viral-host interactions in cancer is progressing, the crosstalk between the tumor and tumor microenvironment still remains to be fully elucidated. In fact, studies on this crosstalk have recently become relevant with the emergence of SARS-CoV-2 infection and its relationship with immunity to influenza A viruses. Of great interest, individuals previously infected with mild non-hospitalized SARS-CoV-2 presented strong innate and adaptive immune responses when given influenza vaccine, and there was also a sex-dimorphic effect with males exhibiting a greater response to influenza vaccine than females (39).

In conclusion, our studies of LUAD cells provide examples of how genetic alterations acquired during lung tumorigenesis can dramatically affect the susceptibility of tumor cells to influenza virus infection and replication, and these differences are associated with changes in interferon pathway responses. We argue that the evaluation of the clinical consequences of respiratory pathogen infection of lung cancer patients will differ according to the responses of their cancer cells to the invading pathogen, and understanding these consequences is critical for appropriate personalized treatment. Additionally, alterations or loss of interferon signaling in cancer cells and susceptibility to viral infections suggest that immune checkpoint blockade antibodies may be less effective in these patients. Thus, these patients may be considered for more effective therapeutic approaches, including targeted therapies, standard chemotherapy, and/or radiation therapy, thereby avoiding the potential side effects of immunotherapy, such as life-threatening cytokine storms.

## MATERIALS AND METHODS

### Cell lines

Human lung adenocarcinoma epithelial cells (A549) from ATCC (American Type Culture Collection) were maintained in high-glucose Dulbecco’s Modified Eagle Medium (DMEM; Corning or Gibco), 10% fetal bovine serum (FBS) (Sigma or Peak Serum), and 100 units/mL Pen/Strep (Corning or Gibco) at 37°C with 5% CO_2_. NCI-H820 and NCI-H322 cell lines were obtained from the laboratory of John Minna at UT Southwestern Medical Center. These cell lines were maintained in RPMI-1640 (Sigma or Gibco), 5% FBS, and 100 units/mL Pen/Strep antibiotics at 37°C with 5% CO_2_. Human embryonic kidney (HEK) 293T cells and MDCK cells were purchased from ATCC and cultured in DMEM (Corning) containing 10% fetal bovine serum (Peak) and 1% penicillin-streptomycin (Corning).

### Antibodies and reagents

For immunofluorescence staining, anti-influenza A virus NP antibody (HT103), anti-mouse IgG Alexa Fluor 488 secondary antibody (Invitrogen, A-11029), and either Hoechst 33258 (Molecular Probes/Life Technologies) or 4’,6-diamidino-2-phenylindole (DAPI, Invitrogen, D1306) were used for DNA visualization. Cytokines used in this study include human IFN-α 2a (Alpha A, PBL Assay Science, 11101-2) and human IFN-λ 1 (PBL Assay Science, 11826-1). Primary antibodies used for western blot include anti-phospho-STAT1 (Tyr701, 58D6, Cell Signaling, 9167S), anti-STAT1 (STAT1-79, Invitrogen, AHO0832), anti-phospho-STAT2 (Tyr690, D3P2P, Cell Signaling, 88410S), anti-STAT2 (Invitrogen, 44-362G), anti-phospho-NF-κB p65 (Ser536, Cell Signaling, 3031S), anti-NF-κB p65 (D14E12, Cell Signaling, 8242S), anti-phospho-IRF-3 (Ser396, 4D4G, Cell Signaling, 4947S), anti-IRF-3 (D6I4C, Cell Signaling, 11904S), anti-IRF-1 (D5E4, Cell Signaling, 8478S), anti-GAPDH (14C10, Cell Signaling, 3683S), anti-influenza A virus M1 (Fig. 5; GeneTex, GTX127356), anti-Influenza A virions (Fig. 1; Meridian Life Science, B65141G), anti-influenza NS1 protein antibody (GTX125990, GeneTex), CH25H (ABS562, EDM Millipore), anti-IFITM1 (Proteintech, 11727-3-AP), anti-IFITM2 (Proteintech, 12769-1-AP), anti-IFITM3 (Life Technologies PA511274), anti-NCOA7 (Proteintech, 23092-1-AP), and anti-β-actin (Sigma, A5441). Horseradish peroxidase (HRP)-conjugated secondary antibodies for Fig. 1 and 7 include donkey anti-rabbit, sheep anti-mouse (GE Healthcare NA934V and NA931V, respectively), and donkey anti-goat (Jackson Immunoresearch 705-035003). Secondary antibodies used for western blots in Fig. 5 were goat anti-rabbit IgG (H + L, HRP, Invitrogen, 31460) and goat anti-mouse IgG (H + L, HRP, Invitrogen, 31430). Anti-GFP antibody to detect VSV-GFP was obtained from GeneTex (GTX113617).

### Viruses

Influenza A viruses (A/WSN/33) propagation was performed by using a low multiplicity of infection (MOI) in embryonated eggs or in MDCK cells from a virus originally grown from a clonal population. Virus propagated using MDCK cells was amplified at MOI of 0.1–0.001 in infection media consisting of Eagle's minimal essential medium (EMEM) (ATCC), 10 mM HEPES (Gibco), 0.125% bovine serum albumin (BSA; Gibco), and 0.5 µg/mL tosyl phenylalanyl chloromethyl ketone (TPCK) trypsin (Worthington Biomedical Corporation). The initial infection was performed by incubating cells with the virus for 1 h at 37°C, followed by washing the cells with PBS and incubation in infection media. After 48–72 h, at which point cell death was occurring, the supernatant was collected and centrifuged at 1,000 × g for 10 min to remove cell debris. Virus aliquots were stored at −80°C. Stocks are controlled for appropriate HA/PFU titer ratios and sequenced by RNAseq to confirm the viral genome sequences. VSV-GFP (Indiana strain) (36) was obtained from G. Barber (University of Miami Miller School of Medicine).

### VSV infection and imaging analysis

A549, H322, and H820 cells were infected with VSV-GFP at MOI of 0.1 for 8 h. Cells were then subjected to immunofluorescence microscopy, as we previously reported (14), using anti-GFP antibody (GeneTex GTX113617). The percent of GFP-positive cells was determined for each condition and shown in Fig. 2B.

### Cytotoxicity

All cells were plated in 96-well plates and allowed to adhere to the plate for 48 h. For experiments determining cell death caused by viral infection (Fig. 1C), cells were then incubated with A/WSN/33 or A/WSN/33 ΔNS1 at MOI of 0.1 for 1 h in the following media: A549 cells were incubated in infection media consisting of EMEM (ATCC), 10 mM HEPES (Gibco), 0.125% BSA (Gibco), and 0.5 µg/mL TPCK trypsin (Worthington Biomedical Corporation), and all other cells were incubated in RPMI 1640 containing 2% FBS and 0.5 µg/mL TPCK trypsin (Worthington Biomedical Corporation). Cells were incubated for 24 h, 48 h, or 72 h before determining ATP levels.

For experiments determining the cytotoxicity of ruxolitinib, H820 cells were allowed to adhere to the 96-well plate for 48 h. After 48 h, cells were washed once with PBS, then incubated in infection media consisting of EMEM (ATCC), 10 mM HEPES (Gibco), 0.125% BSA (Gibco), 0.5 µg/mL TPCK trypsin (Worthington Biomedical Corporation), 2% FBS, and either 0.05% DMSO or 10 µM ruxolitinib (MedChemExpress, HY-50856). After 24 h and 48 h incubation, ATP levels were measured. For wells used for the 48 h timepoint, media were removed after 24 h and replaced with fresh media with either 0.05% DMSO or 10 µM ruxolitinib. Cytotoxicity was assessed using CellTiter-Glo (Promega), according to the manufacturer’s protocol.

### Single-molecule RNA fluorescence *in situ* hybridization

Cells were infected with influenza A/WSN/33 virus at MOI of 2 for 8 h and then fixed with 4% paraformaldehyde for 15 min. Cells were kept overnight (16 h) in 70% ethanol at 4°C. Cells were then washed in PBS and incubated for 5 min in a wash buffer consisting of Nuclease Free Water, 2× saline-sodium citrate (SSC) Buffer (Sigma), and 10% formamide (Sigma). Probes for the viral M mRNA (14) or oligo-d(T) probe (40) were hybridized by incubation with the cells for 4 h at 37°C in wash buffer containing 10% dextran sulfate and followed by incubation in wash buffer for 30 min at 37°C. Cells were washed twice in PBS for 5 min followed by staining with 1 µg/mL Hoechst 33258 (Molecular Probes/Life Technologies) for 10 min. Cells in 384-well plates were kept in PBS until imaging.

### Imaging analysis

Imaging and quantification for percent infection in Fig. 1 were performed in the University of Texas Southwestern Medical Center High Throughput Screening Core facility. The IN Cell Analyzer 6000 (Molecular Devices, San Jose, CA) was used to image samples. Multiple widefield images were taken per well using a Nikon 20×/0.45 objective with 405, 488, and 561 nm excitation lasers and corresponding emission filters. Image analysis was performed using CellProfiler 4.0.7. Individual nuclei were segmented in the DNA channel and used as seeds to find the extent of the cell body in the poly(A) channel. Individual cell objects were then tagged as infected or uninfected using a custom workflow in Pipeline Pilot Professional 2018 (Dassault Systèmes Biovia). Cells were marked as infected if their M1 signal was equal to or higher than the 99th percentile M1 intensity measurement of the uninfected control wells. The workflow then calculated for each well the total number of cells, the number of infected cells, and the M1 signal for infected cells.

### CNV analysis

*DNA copy number analysis*: copy number variation was estimated from whole-exome sequencing (dbGaP Study Accession: phs001823.v1.p1; https://www.ncbi.nlm.nih.gov/projects/gap/cgi-bin/study.cgi?study_id=phs001823.v1.p1) using the R package “DNAcopy” with the following adjustments: *choice of diploid controls*: because of differences in target capture enrichment during whole-exome sequencing, diploid controls needed to be selected from the same batch as tumor samples. *Recalibration*: because tumor samples are generally not diploid overall (i.e., they do not have an equal amount of copy number gains and losses), copy numbers were recalibrated by visual inspection of each DNAcopy-generated plot so that areas of the genome with two copies should have log_2_(tumor/control) = 0.

### Dose-response curves with interferons during infection

Cells were seeded in 96-well black/clear-bottom assay plates and incubated overnight at 37°C with 5% CO_2_ to attain a final cell density of 7,000 cells/well. Following incubation, cells were infected with A/WSN/33 at MOI of 0.1 in the infection medium and treated with twofold serial dilutions of a cytokine of interest at the time of infection. Concentrations of IFN α-2a and IFN-λ 1 ranged from 3,200 pg/mL to 0 pg/mL (Fig. 6) or 1,000 ng/mL to 0 ng/mL (Fig. S1). During infection, treatment (36 h or 48 h), and mock conditions, cells were maintained in the infection medium composed of OptiPRO SFM medium (Gibco) supplemented with 2× GlutaMAX (Invitrogen), 0.5 µg/mL amphotericin B (Sigma-Aldrich), and 1% penicillin/streptomycin (Corning). Each condition was performed in triplicates. Assay plates were further incubated for 36 h or 48 h at 37°C in 5% CO_2_ and then processed for immunofluorescence staining. Briefly, cells were fixed with 10% methanol-free formaldehyde (Polysciences), permeabilized with 0.1% Triton X-100 (Fisher Scientific) in PBS (Corning) for 15 min, blocked in PBS with 0.1% Tween-20 (PBS-T) containing 3% BSA for 1 h, and immuno-stained with anti-influenza A virus NP antibody (HT103) at a 1:500 dilution in PBS-T containing 3% BSA for 1 h, followed by another 1 h incubation with both anti-mouse IgG Alexa Fluor 488 secondary antibody at a 1:1,000 dilution and DAPI at a 1:2,000 dilution in PBS-T containing 3% BSA. Percent inhibition was determined using the Celigo Image Cytometer (Nexcelom Bioscience).

### JAK inhibitor treatment

H820 cells were allowed to adhere to coverslips in 24-well plates for 48 h. After 48 h, cells were washed once with PBS, then incubated in infection media consisting of EMEM (ATCC), 10 mM HEPES (Gibco), 0.125% BSA (Gibco), 0.5 µg/mL TPCK trypsin (Worthington Biomedical Corporation), 2% FBS, and either 0.05% DMSO or 10 µM ruxolitinib (MedChemExpress, HY-50856). After 1 h treatment, cells were then mock infected with A/WSN/33 at MOI of 0.1 or 1 in infection media with DMSO alone or ruxolitinib for 1 h. After 1 h incubation with virus, media containing virus were removed and replaced with 0.5 mL of fresh media with either 0.05% DMSO or 10 µM ruxolitinib. For 36 h and 48 h timepoints, 0.5 mL additional fresh media with either 0.05% DMSO or 10 µM ruxolitinib was added to the wells at 18 h or 24 h, respectively. After 18 hpi, 36 hpi, and 48 hpi, cells were fixed with 4% paraformaldehyde for 15 min before performing smRNA-FISH as described above.

### Real-time reverse transcription PCR (RT-qPCR)

Cells were seeded in 24-well plates and incubated at 37°C, 5% CO_2_, for 48 h. Cells were then either mock-infected, infected with influenza virus at an MOI of 1, mock-treated, or treated with a cytokine of interest at 50 pg/mL or 25 pg/mL. Each condition was performed in triplicates. Assay plates were further incubated overnight at 37°C in 5% CO_2_. Cells were lysed, and total RNA was isolated using RiboPure RNA purification kit (Invitrogen) according to the manufacturer’s instructions. Isolated RNA was then reverse transcribed using a high-capacity cDNA reverse transcription kit with RNase inhibitor (Applied Biosystems) according to the manufacturer’s instructions. For each sample, reactions were prepared in duplicates for qPCR using LightCycler 480 SYBR Green I master mix (Roche). Primers targeting each human or viral gene of interest were used at a final concentration of 1 µM (Table 1). qPCR was performed using LightCycler 480 Instrument II (Roche), where the cycling program was designed according to the manufacturer’s instructions and set to 50 amplification cycles.

**TABLE 1:** Primers used for qPCR

Target gene	Forward primer sequence (5′–3′)	Reverse primer sequence (5′–3′)
*IFN α−2a*	TGGGCTGTGATCTGCCTCAAAC	CAGCCTTTTGGAACTGGTTGCC
*IFNß*	TCTGGCACAACAGGTAGTAGGC	GAGAAGCACAACAGGAGAGCAA
*IFN-λ1*	AACTGGGAAGGGCTGCCACATT	GGAAGACAGGAGAGCTGCAACT
*MX1*	GTGGCTHAHAACAACCTGTG	GGCATCTGGTCACGATCCC
*GAPDH*	GTCTCCTCTGACTTCAACAGCG	ACCACCCTGTTGCTGTAGCCAA
*M1*	AGATGAGTCTTCTAACCGAGTCG	TGCAAAAACATCTTCAAGTCTCT

### RNA-seq and data analysis

H322 and H820 cells were infected at MOI of 1 for 8 h in infection media consisting of EMEM (ATCC), 10 mM HEPES (Gibco), 0.125% BSA (Gibco), 0.5 µg/mL TPCK trypsin (Worthington Biomedical Corporation), and 2% FBS. Total RNA was collected using the RNeasy Plus Mini Kit (Qiagen). RNA sequencing data were trimmed using Trimmomatic (41), and filtered sequences were aligned to HRch38 using the Ensembl genome. Raw counts were obtained from Binary Alignment Map (BAM) files using the Rsubread package (42). Additional analysis was performed using R version 4.2.0 and Bioconductor 3.15 in RStudio (R Core Team, 2020; RStudio Team, 2020). Differential expression analysis between conditions was determined using the DESeq2 package (43) with Benjamini–Hochberg correction (false discovery rate <0.05) (44). Both gene set enrichment analysis of the Kyoto Encyclopedia of Genes and Genomes (KEGG) database and overexpression analysis using the Gene Ontology (GO) Biological Processes database were performed using the clusterProfiler package (45).

### Western blot

For Fig. 1E and 7G, H322 and H820 cell lines were plated for 48 h at 37°C in 5% CO_2_. Cells were then mock-infected, infected with wild-type A/WSN/33, or infected with A/WSN/33ΔNS1 at MOI of 2 for 8 h in infection media consisting of EMEM (ATCC), 10 mM HEPES (Gibco), 0.125% BSA (Gibco), 0.5 µg/mL TPCK trypsin (Worthington Biomedical Corporation), and 2% FBS. Cells were lysed in 2× sample buffer (125 mM Tris HCl pH 6.8, 20% glycerol, and 4% SDS), boiled for 10 min, sonicated, and then boiled again for 10 min. Protein concentration was measured with the Biorad DC Protein Assay Kit. Protein samples were mixed with dithiothreitol (final concentration 0.125 mM) and bromophenol blue (final concentration 0.0025%) before running on a Bolt Bis-Tris Plus precast polyacrylamide gel (Invitrogen) using Bolt MES SDS Running Buffer (Invitrogen). Proteins were then transferred to a polyvinylidene difluoride (PVDF) membrane (Millipore) using 1× Tris-Glycine buffer (Bio-Rad Laboratories). Membranes were blocked for 1 h at room temperature in 5% non-fat dry milk (Blotting-Grade Blocker, Bio-Rad Laboratories) in Tris-buffered saline (TBS) containing 0.05% Tween-20 (TBS-T). Primary antibodies were diluted using TBS-T with 5% BSA, and secondary HRP antibodies were diluted 1:10,000 in TBS-T with 5% non-fat dry milk. Primary and secondary antibody dilutions were each incubated at room temperature for 1 h. For HRP signal, membranes were incubated with enhanced chemiluminescence (ECL) reagent (Pierce, Thermo Scientific) for 5 min at room temperature.

For Fig. 5D, cells were seeded in 12-well plates at a density of 250,000 cells/well and then incubated at 37°C in 5% CO_2_ overnight. Cells were then either mock-infected and mock-treated, infected with A/WSN/33 at an MOI of 1, or treated with 50 pg/mL of human IFNα-2a. Each condition was performed in triplicates. Assay plates were further incubated for 24 h at 37°C in 5% CO_2_. Cells were lysed in radio-immunoprecipitation assay buffer (Sigma-Aldrich) supplemented with 1% SDS (Invitrogen) and cOmplete EDTA-free protease inhibitor cocktail (Roche). Lysates were normalized for protein concentration using a Pierce BCA protein assay (Thermo Fisher Scientific) supplemented with 4× Laemmli sample buffer (Bio-Rad Laboratories), boiled for 5 min, and loaded into 4%–20% gradient gels (Bio-Rad Laboratories). Gels were transferred to 0.2 µm PVDF membranes (Bio-Rad Laboratories) using a Trans-Blot Turbo Transfer System (Bio-Rad Laboratories). Membranes were blocked for 1 h in TBS with 0.05% Tween-20 (TBS-T) containing 5% BSA. Primary antibodies were diluted 1:1,000, and secondary antibodies were diluted 1:10,000 in TBS-T containing 3% BSA.

### IAV-based BlaM1-VLP production and infection

H1N1-pseudotyped BlaM1-VLPs were generated as previously described (19). Briefly, HEK293T cells were seeded in six-well plates and co-transfected with 2 µg of pCAGGS-A/WSN/33-BlaM1, 0.5 µg of pCAGGS-A/WSN/33-H1, and 0.5 µg of pCAGGS-A/WSN/33-N1 using ViaFect (Promega) at a DNA to ViaFect ratio of 1 to 3. At 6 h post-transfection, supernatants were replaced with Opti-MEM containing 1% penicillin-streptomycin. At 72 h post-transfection, VLPs were harvested and treated with 5 µg/mL of TPCK-trypsin (Sigma-Aldrich, catalog# T8802) for 20 min at 37°C followed by treatment with 10 µg/mL trypsin inhibitor from soybean (ThermoFisher Scientific, catalog# 17075029) for 20 min at 37°C. For infection with H1N1-pseudotyped BlaM1-VLPs, 100,000 cells were seeded in 24-well plates the day before the experiment. Cells were infected with 200 µL of VLPs diluted in Opti-MEM for 4 h at 37° in the presence of 0.1 µg/mL DEAE-Dextran hydrochloride (ThermoFisher, catalog# ICN19513380). Cells were collected by trypsinization and incubated with LiveBLAzer FRET-B/G Loading Kit with CCF2-AM (ThermoFisher Scientific, catalog# K1032) for 30 min at 37°C. Samples were analyzed by flow cytometry, and dead cells were excluded using LIVE/DEAD Fixable Near-IR Dead Cell Stain Kit (ThermoFisher Scientific). Entry-positive cells were determined using FlowJo v10 software by gating on events with cleaved CCF2-AM.

### IAV entry assay and immunofluorescence

To assess influenza virus entry, A549, H322, and H820 cells were plated on coverslips coated with 0.2% gelatin. After 48–72 h, plates with coverslips were placed on ice for 10 min. Cells were then washed with cold PBS and mock-infected or infected with A/WSN/33 at MOI of 5 in cold infection media for 45 min. Cells were then washed with cold PBS followed by incubation at 37°C in infection media containing EMEM (ATCC), 10 mM HEPES (Gibco), 0.125% BSA (Gibco), 0.5 µg/mL TPCK trypsin (Worthington Biomedical Corporation), 2% FBS, and cycloheximide at 500 µM for H322 and H820 cells or 1 mM for A549 cells for 2 h or 5 h.

To follow influenza virus entry by staining the viral NP protein, cells were fixed with 4% paraformaldehyde in PBS for 15 min at room temperature, then permeabilized in 0.5% Triton X-100 in PBS for 5 min, and finally washed three times in PBS for 5 min. Coverslips were blocked with 5% BSA in PBS for 1 h, then incubated with NP (HT103) antibody in PBS containing 5% BSA for 1 h at room temperature. Coverslips were washed three times in PBS for 5 min each time, followed by incubation with anti-mouse IgG Alexa Fluor 488 secondary antibody in PBS containing 5% BSA for 1 h at room temperature. Coverslips were then washed once with PBS for 5 min, then incubated with Hoechst in PBS (1 µg/mL) for 10 min and washed with PBS for 5 min. Coverslips were mounted on glass slides with ProLong Diamond Antifade Mountant (Invitrogen). Images were analyzed in a Nikon Eclipse Ti2 microscope using NIS-Elements software. Images were captured with a Nikon Plan Apochromat λD oil immersion lens (magnification = 60× and NA = 1.4) and a Teledyne BSI Prime Express camera (resolution = 0.11 µm/px). For each image, 15 *z* planes with a separation of 0.3 µm were taken. Each image was deconvolved using a blind 3D algorithm in AutoQuant X3. The percentage of cells that showed NP fluorescence staining was determined for each cell type (A549, H322, and H820 cells), mock-infected or infected, and the results are shown in the histograms in Fig. 7F. A549 cells were used as a reference for the percentage of NP-positive cells in this assay (Fig. 7C and F).

## Data Availability

RNA-seq analysis of total RNA from lung adenocarcinoma cell lines non-infected or infected with influenza virus is deposited in the SRA database with the following accession number: PRJNA1186930.

## SUPPLEMENTAL MATERIAL

Supplemental material jvi.00469-25-s0001.pdf Legend for Table S1; Fig. S1 and S2.

Table S1 jvi.00469-25-s0002.xlsx RNAseq analysis of H322 and H820 cells shown in Fig. 4A.

ASM does not own the copyrights to Supplemental Material that may be linked to, or accessed through, an article. The authors have granted ASM a non-exclusive, world-wide license to publish the Supplemental Material files. Please contact the corresponding author directly for reuse.
