# Supplementary material for: Contrasting interferon-mediated antiviral responses in human lung adenocarcinoma cells
Source: J Virol. 2025 May 28;99(6):e00469-25. doi: 10.1128/jvi.00469-25 (PMC12172473; doi:10.1128/jvi.00469-25)
Supplement: Supplemental material — Legend for Table S1; Fig. S1 and S2. [file jvi.00469-25-s0001.pdf]

SUPPLEMENTAL MATERIAL

Contrasting Interferon-Mediated Antiviral Responses in Human Lung Adenocarcinoma Cells

Matthew Esparza[1*], Sara S. El Zahed[2,3*], Umut Karakus[2,3], Hanspeter Niederstrasser[4], Boning Gao[5,6], Kimberly Batten[1], Jerry W. Shay[1], Bruce Posner[4], Fred R. Hirsch[7,8,9], Luc Girard[5,6], Lily Jun-shen Huang[1], John Minna[6,10], Adolfo García-Sastre[2,3,8,9,11,12#], and Beatriz M. A. Fontoura[1#]

[1]Department of Cell Biology, University of Texas Southwestern Medical Center, Dallas, TX 75390, USA

[2]Department of Microbiology, Icahn School of Medicine at Mount Sinai, New York, NY 10029, USA

[3]Global Health and Emerging Pathogens Institute, Icahn School of Medicine at Mount Sinai, New York, NY 10029, USA

[4]Department of Biochemistry, University of Texas Southwestern Medical Center, Dallas, TX 75390, USA

[5]Department of Pharmacology, University of Texas Southwestern Medical Center, Dallas, TX 75390, USA

[6]Hamon Center for Therapeutic Oncology Research, University of Texas Southwestern Medical Center, Dallas, TX 75390, USA

[7]Center for Thoracic Oncology, Icahn School of Medicine at Mount Sinai, New York, NY 10029, USA

[8]The Tisch Cancer Institute, Icahn School of Medicine at Mount Sinai, New York, New York, USA

[9]Department of Pathology, Molecular and Cell-Based Medicine, Icahn School of Medicine at Mount Sinai, New York, New York, USA

[10]Departments of Internal Medicine and Pharmacology, University of Texas Southwestern Medical Center, Dallas, TX 75390, USA

[11]Department of Medicine, Division of Infectious Diseases, Icahn School of Medicine at Mount Sinai, New York, New York, USA

[12]The Icahn Genomics Institute, Icahn School of Medicine at Mount Sinai, New York, New York, USA

[*]Equal contribution. Order was determined by the primary discovery.

[#]Corresponding authors: Adolfo García-Sastre (email: adolfo.garcia-sastre@mssm.edu) and Beatriz M. A. Fontoura (e-mail: beatriz.fontoura@utsouthwestern.edu)

Supplemental Table S1 legend and Supplemental Figures

Table S1. RNAseq analysis of H322 and H820 cells shown in Figure 4A. (**Tab 1)** 469 differentially expressed mRNAs between H322 and H820 cells infected with A/WSN/33. Calculated log2FoldChange is fold change of H820 over H322 mRNA levels. (**Tab 2**) From the 469 mRNAs in Tab 1, 340 mRNAs are hits in the Influenza A Virus (IAV) Meta Database. (**Tab 3**) 339 mRNAs that encode restriction factors. (**Tab 4**) Overlap of mRNAs listed as host factors by the IAV Meta Database from Tab 2 (340 mRNAs) and hits in the Interferome database from Tab 4. All these mRNAs listed are hits in the IAV Meta Database while mRNAs highlighted in green are hits in the Interferome database. (**Tab 5**) mRNAs that were not identified as host factors in the IAV Meta Database. mRNAs highlighted in green are hits in the Interferome database. (**Tab 6**) Constitutive levels of IFNs differentially expressed in H820 versus H322 cells.

Figure S1

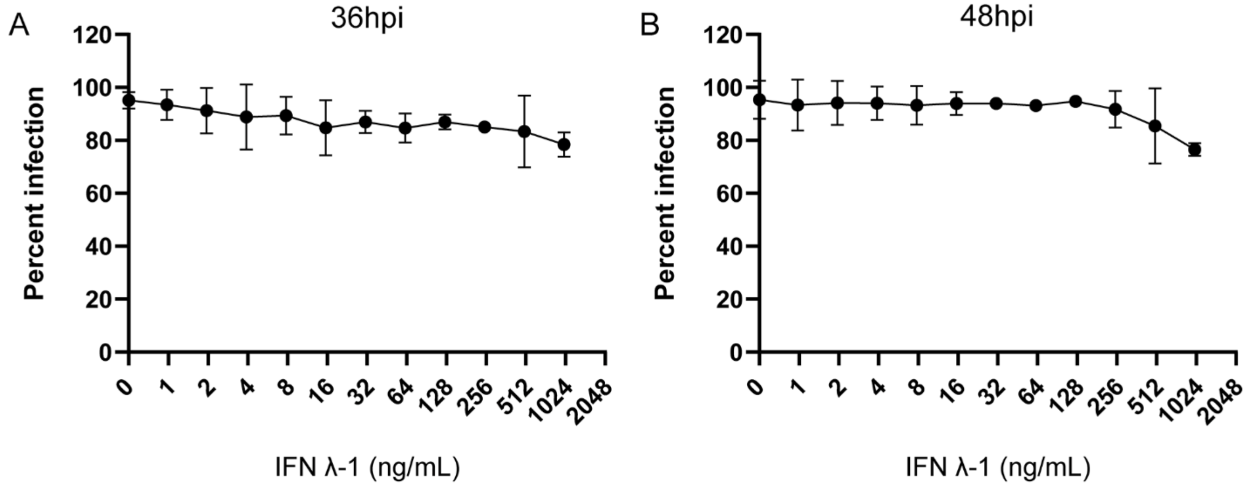

Figure S1. Percent infection of A549 cells with increasing concentrations of interferon-λ1. A549 cells were infected with A/WSN/33 at 0.1 MOI and percent infection was determined in the presence of increasing concentrations of human interferon-λ1 at 36 h (**A**) or 48 h (**B**) post-infection. Interferon treatment started at the time of infection. Graphs show mean +/- SD.

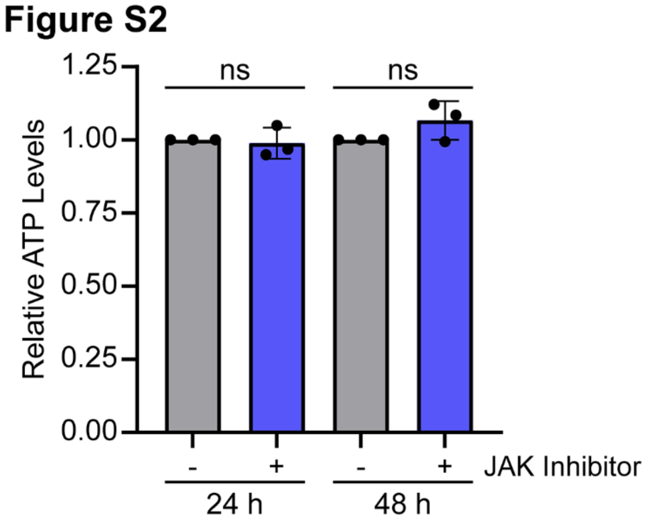

Figure S2. Treatment of ruxolitinib does not alter cell viability in H820 cells. Relative ATP levels of cells treated with 10 mM ruxolitinib compared to DMSO control treated cells. Graphs show the relative ATP levels determined by Cell Titer Glo. Bars represent means +/- SD and each point represents a single replicate, *n=3*. ns, not significant. *p* values were calculated using unpaired two-tailed Student's t test.
